# Biomechanical stability of complex coronal plane fracture fixation of the capitellum

**DOI:** 10.1007/s00402-021-04126-1

**Published:** 2021-08-23

**Authors:** Paul Borbas, Rafael Loucas, Marios Loucas, Maximilian Vetter, Simon Hofstede, Lukas Ernstbrunner, Karl Wieser

**Affiliations:** 1 Department of Orthopedics, Balgrist University Hospital, Forchstrasse 340, 8008 Zurich, Switzerland; 2 Institute of Biomechanics, ETH Zurich, Zurich, Switzerland

**Keywords:** Distal humerus, Capitellum, Complex coronal plane fracture, Antiglide plate, Locking plate, Biomechanics

## Abstract

**Introduction:**

Coronal plane fractures of the distal humerus are relatively rare and can be challenging to treat due to their complexity and intra-articular nature. There is no gold standard for surgical management of these complex fractures. The purpose of this study was to compare the biomechanical stability and strength of two different internal fixation techniques for complex coronal plane fractures of the capitellum with posterior comminution.

**Materials and methods:**

Fourteen fresh frozen, age- and gender-matched cadaveric elbows were 3D-navigated osteotomized simulating a Dubberley type IIB fracture. Specimens were randomized into one of two treatment groups and stabilized with an anterior antiglide plate with additional anteroposterior cannulated headless compression screws (group antiGP + HCS) or a posterolateral distal humerus locking plate with lateral extension (group PLP). Cyclic testing was performed with 75 N over 2000 cycles and ultimately until construct failure. Data were analyzed for displacement, construct stiffness, and ultimate load to failure.

**Results:**

There was no significant difference in displacement during 2000 cycles (*p* = 0.291), stiffness (310 vs. 347 N/mm; *p* = 0.612) or ultimate load to failure (649 ± 351 vs. 887 ± 187 N; *p* = 0.140) between the two groups.

**Conclusions:**

Posterolateral distal humerus locking plate achieves equal biomechanical fixation strength as an anterior antiglide plate with additional anteroposterior cannulated headless compression screws for fracture fixation of complex coronal plane fractures of the capitellum. These results support the use of a posterolateral distal humerus locking plate considering the clinical advantages of less invasive surgery and extraarticular metalware.

**Level of evidence:**

Biomechanical study.

## Introduction

Coronal plane fractures of the humerus that involve the capitellum and the trochlea are rare injuries and account for only 1% of all elbow fractures and 3–4% of all distal humerus fractures [1, 5]. The most common injury mechanism is a low-energy trauma, following a fall from a standing height, on an outstretched hand with a slightly flexed or extended elbow from standing height. This generates a vertical shearing force on the capitellum by the radial head, resulting in a fracture of the capitellum in the coronal plane with or without extension into the trochlea [5, 9, 21].

Due to their small size, inherent intra-articular nature, and propensity to displace, resulting in obstructing elbow motion, these fractures can be challenging to treat. A few diagnostic classification systems have been published to help diagnosing these fractures [1, 5, 7, 10, 15, 16, 32]. The first classification which can guide surgical management and predict outcome was introduced in 2006 by Dubberley et al. [7]: Based on this classification, Type-I fracture involves the capitellum with or without lateral trochlear ridge involvement; Type-II fracture affects the capitellum and extends into the trochlea; and Type-III fracture involves a separate lateral trochlear ridge fragment. Each fracture type was further classified according to the absence (A) or presence (B) of posterior condylar comminution.

As conservative treatment of displaced fractures may lead to complications such as nonunion, chronic pain, rigidity, degenerative arthritis, instability, and a severe compromise in function owing to the restricted range of motion (ROM), operative treatment has been considered as the gold standard [1, 7, 10, 11, 13, 20]. Due to the complexity and inherent intra-articular nature of coronal shear fractures of the capitellum, there is no established gold standard for surgical management of these fractures [25]. Dubberley Type-II fractures are especially challenging to treat because of the fracture extension into the trochlear, and further difficulty is presented when there is associated posterior comminution (Type IIB).

For simple coronal shear fractures of capitellum, the most commonly described technique is individual screw fixation, and several clinical outcome studies reported favorable outcomes[17, 22, 27, 34, 35]. Recent biomechanical studies concluded that simple coronal shear fractures (Dubberley type IA) can be sufficiently stabilized by use of two headless compression screws [3, 8].

In case of comminuted and complex fractures several clinical studies have favored the use of an antiglide plate [26], or a posterolateral locking plate [31]. However, to the author’s knowledge, no biomechanical studies have been performed assessing surgical treatment options in Dubberley Type-II fractures to date. Therefore, we performed this biomechanical study to compare the biomechanical stability of (1) two anteroposterior headless compression screws and an additional anterior antiglide plate (group antiGP + HCS); and (2) posterolateral distal humerus locking plate (group PLP) for Dubberley type IIB coronal shear fractures of the capitellum. We hypothesized that both techniques offer similar and sufficient biomechanical fracture stability.

## Materials and methods

Institutional review board approval was obtained for this cadaveric study.

### Experimental design

A biomechanical study design was established to evaluate the biomechanical stability of two different fracture fixation techniques for complex coronal plane fractures of the capitellum. For the study purposes, 14 fresh-frozen cadaveric hemitorsi (7 left, 7 right) were obtained from Science Care (Phoenix, AZ). The mean age was 78.2 ± 10.4 years at the time of death, and there were six male and eight female donors. As determined by CT-scan, all elbow specimens had intact distal humeri, and further CT-scan evaluation included bone mineral density assessment in a 10 × 10 mm area at the capitellum to ensure comparable bone quality for fracture fixation.

Each group contained randomly assigned specimens, and fracture creation and fixation of the capitellum was conducted in a standardized manner by one fellowship-trained shoulder and elbow surgeon (PB). A standardized testing protocol was used to ensure reproducibility, and all testing were conducted on the same day as the tissue preparation.

### Specimen preparation

Fourteen fresh-frozen cadaveric elbows were thawed for 24 h under room temperature. All elbows were dissected using an anterolateral approach. After removing the skin, superficial tissue, the triceps, extensors and the anconeus muscle, the radiocapitellar joint was identified. Resection was then extended medially until exarticulation of the elbow joint was performed with resection of all soft tissues. Proximally, the humerus was cut 2 cm distal to the deltoid tuberosity. Specimens were kept moist with phosphate-buffered saline to prevent dry out during specimen preparation, surgical repair, and testing.

All elbow specimens underwent a computer-assisted 3D planned osteotomy of the distal humerus for creating a standardized, complex coronal plane fracture of the capitellum with posterior comminution (Dubberley IIB).

### Preoperative planning

Preoperative planning was based on computed tomography (CT) scans of the fresh-frozen cadaveric hemitorsi. All CT scans were performed at our institution, using Siemens Definition AS^®^ or Somatom Edge CT^®^ scanners (Siemens Healthcare GmbH, Eschborn, Germany). Slice thickness was 1.0 mm with an in-plane resolution (x–y) of 0.4 × 0.4 mm. Data were imported by a dedicated application program that enables segmenting the humeral bone using the global thresholding and region growing functionality of a standard segmentation software (Mimics Medical, Materialise NV, Leuven, Belgium) to generate 3D bone models [29]. A Laplacian level-set segmentation growth algorithm advances the outline towards the boundary of the bone. A polygonal mesh was finally extracted, which was used for visualization and planning of the 3D cutting guides.

**Fig. 1:**
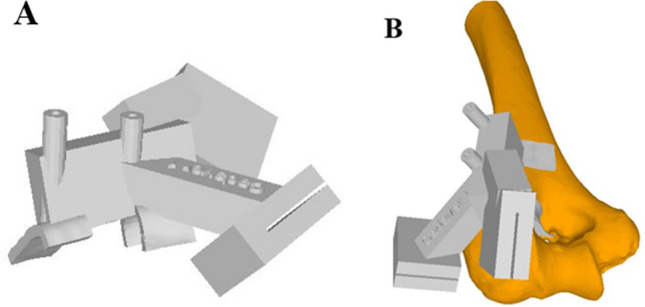
3D printed-patient-specific osteotomy guides. **A** Humerus-specific 3D cutting guide **B** Placement of the cutting guide on the surface of the distal humerus

During the preoperative planning, the surgeon was able to interactively set the cutting plane's position and orientation in the virtual humerus. Thereafter, the models were imported into in-house–developed planning software, CASPA (Balgrist CARD AG, Zürich, Switzerland). A humerus-specific cutting guide was used in all elbow specimens, which snugly fitted to the bone geometry (see Fig. 1A). Based on files provided in the stereolithography format, the biocompatible polyamide cutting guides were 3D printed with a 3D printer (Prusa i3 MK3S kit, Prague, Czech Republic). The guides were used to create a standardized, complex plane fracture of the capitellum with posterior comminution (Dubberley IIB). For this purpose, the guide's body was shaped in a way that it could be uniquely placed by using characteristics of the irregular-shaped surfaces of the distal humerus [19, 29] (Fig. 1B). After registering the fracture situation, the guide position was maintained by placing reference K-wires through drill sleeves connected to the guide body of the primary guide and a cutting slit [19] to constrain the saw blade according to the planned osteotomy plane [29]. The posterolateral column fragment was finally split into two separate fragments after the cuts with the guide were performed.

All 14 elbows were randomly assigned to one of two fracture fixation groups by use of a randomization software, allowing seven age- (within 5 years) and gender-matched distal humeri per group to be tested.

After fracture reduction with the aid of a pointed reduction clamp, temporary fixation was achieved with two 0.8 mm K-wires in each distal humerus of the two fixation groups. An image intensifier was used in all cases during fracture fixation to confirm anatomic reduction before final fixation visually. CT scans were performed after fracture fixation to ensure anatomic fracture fixation and correct screw positioning and length.

### Surgical fracture fixation

#### Group antiGP + HCS

##### Anterior antiglide plate and additional two anteroposterior headless compression screws

**Fig. 2:**
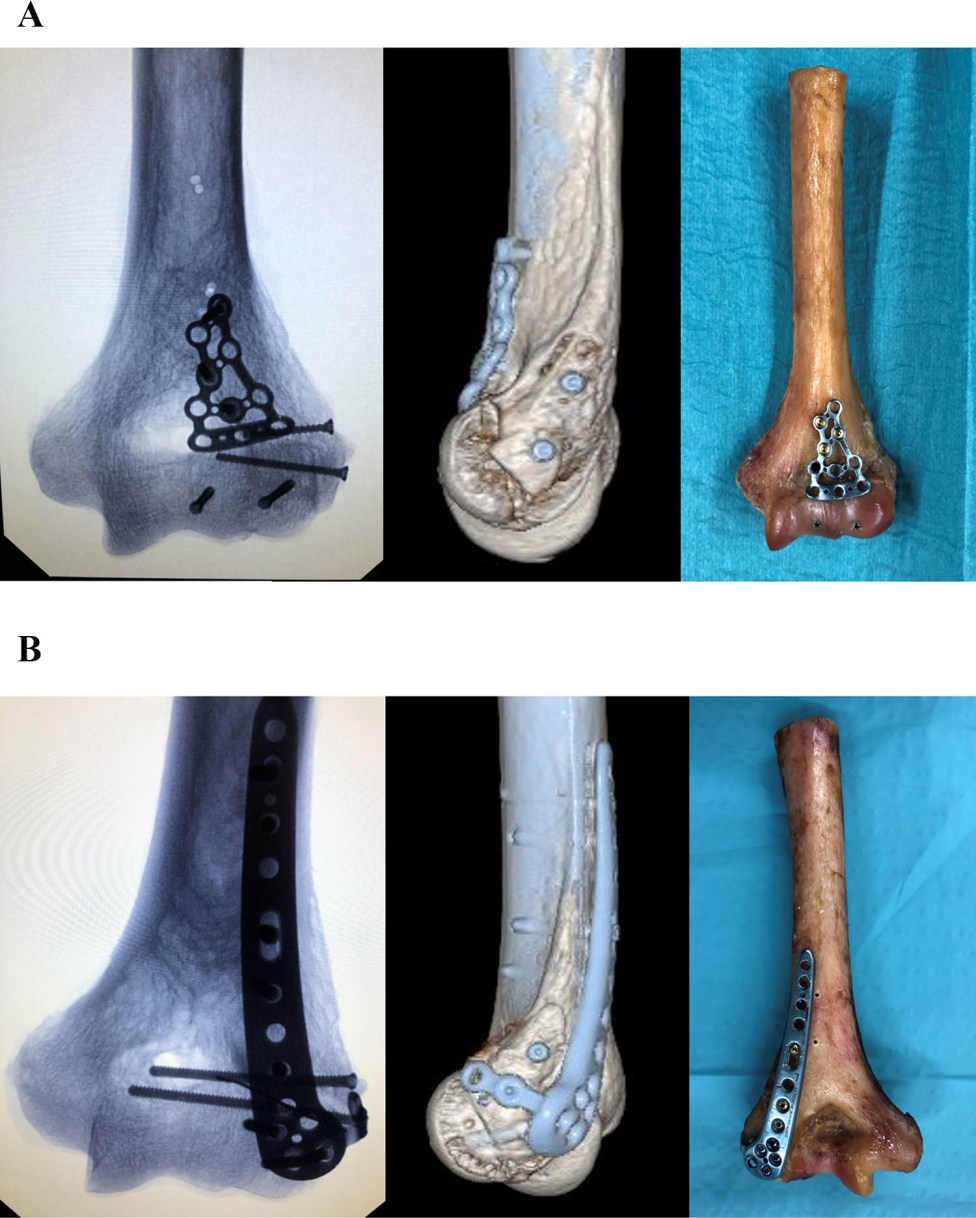
Illustration of fracture fixation of simple coronal shear fractures of the capitellum (Dubberley 2**B**) with two different fixation methods: 2**A**) Anteroposterior x-ray (left), lateral 3D CT scan (middle) and anteroposterior view (right) of two anteroposterior HCSs with additional anterior antiglide plate (group HCS + antiGP). 2**B**) Anteroposterior x-ray (left), lateral 3D CT scan (middle) and posteroanterior view (right) of posterolateral distal humerus locking plate (group PLP)

Two parallel 0.8 mm K-wires were placed in an anteroposterior direction into the capitellum perpendicular to the fracture line with about 1 cm distance in between. Screw length was measured with a cannulated depth gauge and partially threaded HCS with a 2.2 mm diameter (Speedtip CCS 2.2 mm, Medartis, Basel, Switzerland) were used. Screw length was chosen just 2 mm shorter than measured, to avoid penetrating the posterior cortex. The cartilage and subchondral bone of the first cortex was predrilled with a cannulated 1.8 mm drill. The self-drilling and self-tapping cannulated HCS screws were gently introduced with a screwdriver into a final position just about 0.5–1 mm below the cartilage level*.* After placement of two anteroposterior HCS (Speedtip CCS 2.2 mm, Medartis, Basel, Switzerland), additional fracture stabilization was performed using an anterior antiglide plate. Therefore, a 2.0 mm frame plate (Trilock, Medartis, Basel, Switzerland) was adjusted to the specific anatomy of each distal humerus to prevent the proximal dislocation of the capitellum. Care was taken to allow at least 120° of flexion in the radiocapitellar joint. The plate was fixed with three 2 mm bicortical proximal screws, directed in a posterolateral direction to avoid posterior screw impingement in the olecranon fossa. The medial and posteromedial fragments were additionally fixed with two separate 2.8 mm cortical screws placed in a lateromedial direction (Fig. 2A).

#### Group PLP

##### Posterolateral distal humerus locking plate

A posterolateral distal humerus locking plate (Trilock 2.8 mm, Medartis, Basel, Switzerland) was adjusted to the individual anatomy of the distal humerus. Initial fixation was performed with proximal 2.8 mm cortical screws. Afterwards, the fracture was fixed with three posteroanterior locking screws placed into the subchondral bone of the capitellum through the distal holes of the plate, perpendicular to the fracture line. The adequate length of the screws was chosen to a position 1 to 2 mm below the articular cartilage. The medial and posteromedial fragments were additionally fixed with two separate 2.8 mm cortical screws placed in a lateromedial direction, with the distal screw placed through the extension hole of the plate (Fig. 2B).

### Experimental protocol

**Fig. 3:**
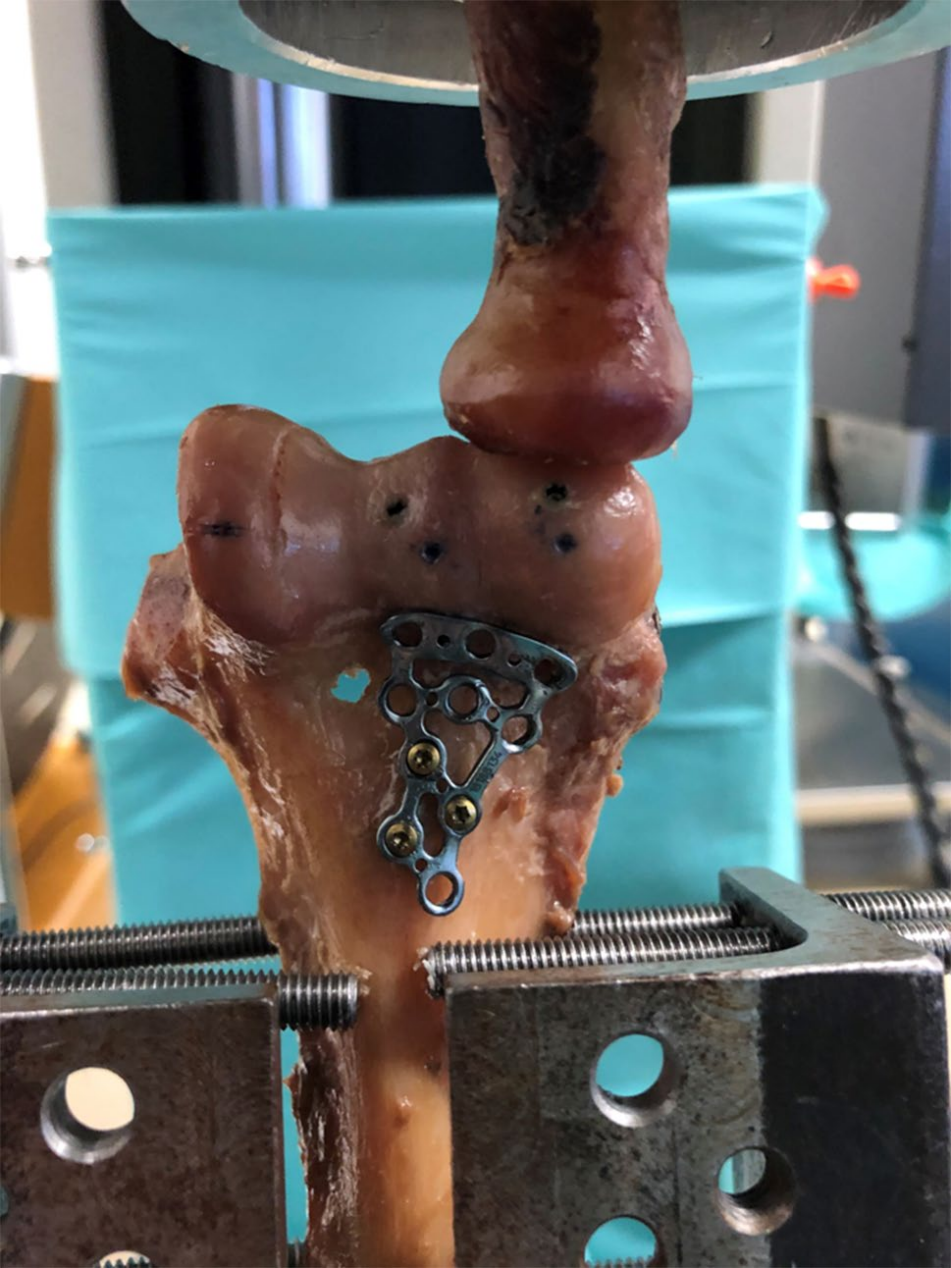
Experimental set-up with loading of the capitellum in 20 degrees elbow flexion of a group HCS + antiGP specimen

**Fig. 4:**
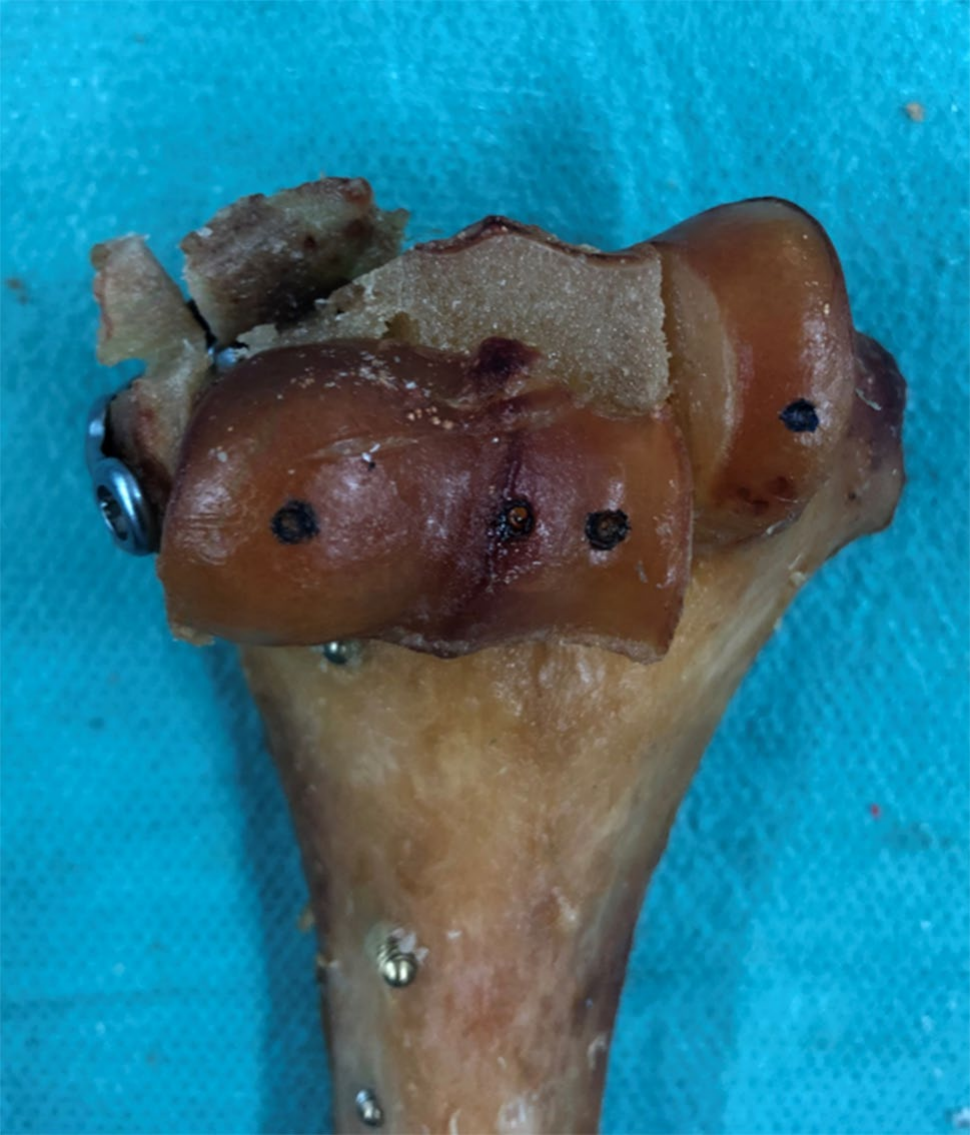
Anterior view of a group PLP specimen after cyclic loading and load to failure testing with the capitellum fragment’s proximal displacement

All biomechanical tests were performed with an uniaxial material testing machine (ZwickRoell GmbH, Ulm, Germany), equipped with a 20 kN load cell. Force (N) and displacement data was recorded with the dedicated software (testXpert v. 10.11, ZwickRoell GmbH, Ulm, Germany).A study protocol similar to the one previously published by Elkowitz et al.[8] was used, with the distal humeri loaded in a position of 20 degrees flexion. This angle was also chosen since it has been previously shown that the greatest amount of force transmitted from the radial head to the capitellum occurs between 0 and 30 degrees of flexion [18]. The proximal radius was centered in a polyvinyl chloride pipe and potted with bone cement before vertically attaching it to the materials testing machine. The resected humeral shaft was fixed in a custom-made jig that allowed for angle adjustments, and was set-up to obtain 20 degrees of flexion. During testing, the load was applied vertically on the capitellum through the distal radius. After a preload of 5 N, specimens were loaded between 5–75 N at 0.5 mm/s for a total of 2000 cycles. Cyclic displacement was evaluated at 90, 180, 360, 900, and 2000 cycles (Fig. 3). Displacement was also measured using a MicroScribe digitizer (MicroScribe MX, Revware Inc., Raleigh, North Carolina, USA) by tracing determined points at the capitellum and trochlea before cyclic loading and after 90, 180, 360, 900, and 2000 cycles. Finally, ultimate load to failure tests were performed at 0.5 mm/s for each specimen. Failure was defined as fragment displacement greater than 3 mm, according to previous biomechanical studies [30] (Fig. 4).

### Statistical analysis

Normal distribution of data was assessed with the Shapiro–Wilk test. Descriptive data were calculated using mean and standard deviation. The two groups were compared with the student’s t-test. Significance was set as *p* < 0.05. A sample size calculation was performed according to the study’s data by Elkowitz et al. [8] using G*Power (Heinrich Heine University, Düsseldorf, Germany), resulting in a sample size of six samples per group to achieve a power > 0.8.

## Results

Mean bone density of the capitellum did not show a statistically significant difference between the two groups (group antiGP + HCS: 255 ± 94 Hounsfield units (HU); group PLP: 288 ± 94 HU; *p* = 0.516).

There was no significant difference in displacement during 90 (*p* = 0.180), 180 (*p* = 0.105), 360 (*p* = 0.200), 900 (*p* = 0.324), and 2000 cycles (*p* = 0.291) under cyclic loading between the two groups. Although not significantly different, the PLP group showed a higher stiffness during 90 (517 vs. 447 N/mm; *p* = 0.345), 180 (537 vs. 457 N/mm; *p* = 0.266), 360 (551 vs. 471 N/mm; *p* = 0.324), 900 (568 vs. 476 N/mm; *p* = 0.270), and 2000 cycles (581 vs. 464 N/mm; *p* = 0.168). Mean total stiffness comparing the two groups was also higher in the PLP group (551 N/mm) compared with the antiGP + HCS group (463 N/mm), but not statistically significant (*p* = 0.253).

The ultimate load to failure was 649 ± 351 HCS + antiGP and 887 ± 187 in the PLP group, again without reaching significant differences (*p* = 0.140).

Detailed results are illustrated in Table 1.

**Table 1:** Displacement and stiffness during cyclic loading as well as ultimate load to failure comparing the two groups

Variable*	HCS + antiGP group	PLP group	*p*†
Displacement 90 cycles, mm	0.4 ± 0.1	0.5 ± 0.2	0.180
Displacement 180 cycles, mm	0.4 ± 0.2	0.6 ± 0.2	0.105
Displacement 360 cycles, mm	0.5 ± 0.2	0.7 ± 0.2	0.200
Displacement 900 cycles, mm	0.6 ± 0.3	0.8 ± 0.2	0.324
Displacement 2000 cycles, mm	0.7 ± 0.3	0.8 ± 0.3	0.291
Stiffness 90 cycles, N/mm	447 ± 124	517 ± 142	0.345
Stiffness 180 cycles, N/mm	457 ± 139	537 ± 118	0.266
Stiffness 360 cycles, N/mm	471 ± 148	551 ± 139	0.324
Stiffness 900 cycles, N/mm	476 ± 155	568 ± 142	0.270
Stiffness 2000 cycles, N/mm	464 ± 160	581 ± 140	0.168
Mean total stiffness, N/mm	463 ± 142	551 ± 132	0.355
Ultimate load to failure⨎, N	649 ± 351	887 ± 187	0.140

*HCS* headless compression screw, *antiGP* antiglide plate, *PLP* posterolateral locking plate

^a^Data are presented as mean ± standard deviation

†The two groups were compared with the one-way analysis of variance (ANOVA; parametric data) and Kruskal-Wallis one-way analysis of variance (non-parametric data)

⨎Ultimate load to failure was defined as fragment displacement >3mm

## Discussion

The present study’s main finding is that both tested stabilization techniques for Dubberley type IIB fractures of the capitellum–an anterior antiglide plate with additional anteroposterior cannulated headless compression screws and a posterolateral distal humerus locking plate—provide similar biomechanical stability. Interestingly, the posterolateral distal humerus locking plate, which is less invasive regarding the approach and extra-articular implant placement [31], had a (non-significant) higher stiffness during cyclic loading over 2000 cycles and a higher ultimate load to failure (887 vs. 649 N).

The type B subdivision of the Dubberley classification refers to posterior comminution in the coronal plane. This classification is helpful in treatment guiding and prediction of the outcome [7]. Limited literature is available on capitellar and trochlear fractures with posterior comminution and their treatment options. Therefore several concerns were previously described: Brouwer et al. reported that posterior comminution of the capitellum and trochlea leads to deterioration in the blood supply of the fragments [4]. Additionally, Ashwood et al. reported the importance of achieving firmness following fracture reduction, thus elbow joint small cartilage blocks should be maintained as required for a reduction and internal fixation during surgery [2]. Jupiter et al. found that restoration of normal anatomy correlates with elbow joint function at the humeral distal frontal plane [12]. Thus, the basic surgical aim of this particular type of fracture’s osteosynthesis is to restore anatomical integrity of the fragments, achieve lateral column stability, prevent interfragmentary rotation, and establish adequate support at the distal humerus [6].

The literature offers numerous studies that report favorable outcomes for the treatment of coronal plane fractures. Anteroposterior headless compression screws demonstrated favorable outcomes for the treatment of coronal plane fractures [8, 15, 17, 23, 28]. However, it is an appropriate technique for simple fractures, but inadequate for the management of Dubberley type B fractures, mostly including multiple fragments at the posterior part of the distal humerus and at the lateral capitellar area where the LCL adheres [6, 33]. Mighell et al. previously reported that if no adequate subchondral bone stock is available, iatrogenic fractures may also occur [17]. Thus, the authors suggested for more stable fixation reconstruction of comminuted coronal plane fractures with a posterolateral plate with posteroanterior fracture fixation or fixation with an anterior antiglide plate with additional anteroposterior cannulated headless compression screws instead of screws alone.

The results of our study are in accordance with recent clinical studies that suggest plating as the treatment of choice in those complex fracture patterns [26, 31]. Wang et al. [31] recently evaluated the safety and efficacy of the fixation of Dubberley type B capitellar and trochlear fractures in a clinical study using posterolateral anatomic plates with support of the distal humerus. The authors concluded that capitellar and trochlear fractures with additional posterior comminution are safely and effectively treated using dorsolateral anatomic plates, resulting in good radiographic and functional outcomes.

Sen et al. introduced the concept of additional fracture stabilization using an anterior placed antiglide plate following traditional AO principles by neutralizing shear forces [24]. Song et al. [26] recently evaluated the technical feasibility and clinical efficacy of coronal shear fractures of the distal humerus using the anti-sliding plate technique. The authors suggest that the anti-sliding plate technique allows a stable internal fixation of the fracture, which is critical for early mobilization and a good functional outcome. Interestingly, our study clearly demonstrates that fracture fixation with an antiglide plate and two additional anteroposteriorly placed HCSs could not outperform a posterolateral plate at all. A posterolateral plate fixation provides sufficient primary stabilization, supporting the use of a posterolateral distal humerus locking plate especially when considering the clinical advantages of less invasive surgery and extraarticular metalware.

The present study has some limitations that should be mentioned. Our biomechanical investigations were performed in only one position of the radiocapitellar joint (in 20° of flexion). Therefore, conclusions are limited concerning the full range of motion as well as the influence of the ulnohumeral joint. However, the position tested was chosen according to previous biomechanical studies, as the highest amount of force transmitted from the radial head to the capitellum occurs within 0–30° of flexion [18]. Second, this study did not consider varus and valgus moments on fracture fixation, although, in varus positioning, radiocapitellar loading decreases and is more pronounced in the ulnohumeral joint [14].

On the other hand, controlled matching (including age and bone quality) and especially using 3D printed guides to create standardized and patient-specific complex coronal plane fractures increases the value and reproducibility of this study.

## Conclusions

Posterolateral distal humerus locking plate osteosynthesis achieves biomechanical equally fixation strength as an anterior antiglide plate with additional anteroposterior cannulated headless compression screws for fracture fixation of complex coronal plane fractures of the capitellum. Fracture fixation with a posterolateral distal humerus locking plate demonstrated a statistically non-significant higher stiffness and ultimate load to failure. These results support the use of a posterolateral distal humerus locking plate for this fracture type considering the clinical advantages of less invasive surgery and extraarticular metalware.
